# Leptospirosis-associated Severe Pulmonary Hemorrhagic Syndrome, Salvador, Brazil

**DOI:** 10.3201/eid1403.071064

**Published:** 2008-03

**Authors:** Edilane L. Gouveia, John Metcalfe, Ana Luiza F. de Carvalho, Talita S.F. Aires, José Caetano Villasboas-Bisneto, Adriano Queirroz, Andréia C. Santos, Kátia Salgado, Mitermayer G. Reis, Albert I. Ko

**Affiliations:** * Fundação Oswaldo Cruz, Ministério da Saúde, Salvador, Bahia, Brazil; † University of California, San Francisco, California, USA; ‡ Hospital Couto Maia, Secretaria da Saúde do Estado da Bahia, Salvador, Bahia, Brazil; § Weill Medical College of Cornell University, New York, New York, USA

**Keywords:** Leptospirosis, Leptospira, pulmonary hemorrhage, urban epidemics, incidence, epidemiology, serovar Copenhageni, dispatch

## Abstract

We report the emergence of leptospirosis-associated severe pulmonary hemorrhagic syndrome (SPHS) in slum communities in Salvador, Brazil. Although active surveillance did not identify SPHS before 2003, 47 cases were identified from 2003 through 2005; the case-fatality rate was 74%. By 2005, SPHS caused 55% of the deaths due to leptospirosis.

Leptospirosis, a spirochetal zoonotic disease, is increasingly recognized as an important cause of hemorrhagic fever (*1*–*4*). The classic presentation of severe leptospirosis, Weil’s disease, is characterized by jaundice, acute renal failure, and bleeding. However, the 1995 Nicaragua outbreak raised awareness for leptospirosis as the cause of a severe pulmonary hemorrhagic syndrome (SPHS) (*5*). This syndrome, first identified in South Korea and People’s Republic of China (*6*), is now reported worldwide (*2*). SPHS is associated with fatality rates of >50% and in certain settings, has replaced Weil’s disease as the cause of death among leptospirosis patients (*7*–*10*).

The factors responsible for SPHS and its emergence are not well understood. In Brazil, outbreaks of leptospirosis occur annually in slum communities during seasonal periods of heavy rainfall (*11*). Although SPHS is a frequently observed manifestation of leptospirosis in Rio de Janeiro and São Paulo (*7*,*8**,**12*), the occurrence of SPHS varies according to geographic region. In the city of Salvador (population 2.7 million), active surveillance did not detect SPHS among 1,786 leptospirosis cases identified from 1996 through 2002 (unpub. data). However, in 2003 we identified a patient in whom massive pulmonary hemorrhage and acute respiratory distress syndrome developed; subsequently, the number of cases with similar manifestations unexpectedly increased. We report the investigation of the emergence of SPHS in a setting where it was not previously observed.

## The Study

The Oswaldo Cruz Foundation and State Secretary of Health of Bahia have conducted active surveillance for leptospirosis since March 1996 in the metropolitan region of Salvador. According to health secretary protocols, suspected cases are referred to the state infectious disease hospital. The study team consecutively identified case-patients who were admitted to this hospital and met the clinical definition for severe leptospirosis (*11*). Study participants were enrolled according to informed consent protocols approved by the Oswaldo Cruz Foundation and Weill Medical College of Cornell University. After the first case was identified on May 1, 2003, the study team prospectively identified SPHS from October 2003 through December 2005 by evaluating patients 5 days a week for findings of massive pulmonary hemorrhage (hemoptysis >300 mL or aspiration of fresh blood after endotracheal intubation, which did not clear with suctioning) and respiratory insufficiency (respiratory rate >30 per min or use of supplemental oxygen therapy). Medical records of patients hospitalized between January 2000 and September 2003 were reviewed to identify cases that may not have been previously recognized.

A data-entry form was used to extract information from medical charts. Patients or family members were interviewed to obtain information on demographics and risk exposures in the household and workplace. Blood samples were collected during hospital admission, on day 4 or 5 of hospitalization, and 2 weeks after the initial sample was obtained. Laboratory-confirmed diagnosis of leptospirosis was defined as a 4-fold rise in titer between paired samples or a single titer of >800 in the microscopic agglutination test (MAT) (*11*) or a positive result on the immunoglobulin M (IgM) ELISA (Bio-Manguinhos, Rio de Janeiro, Brazil) (*13*). The MAT panel included the 19 reference strains recommended by the World Health Organization (Royal Tropical Institute, the Netherlands) and a clinical isolate, *Leptospira interrogans* serovar Copenhageni strain Fiocruz L1–130 (*11*).

Surveillance identified 47 (10%) SPHS cases among 474 patients who met the clinical definition for severe leptospirosis from 2003 through 2005. Review of medical records did not identify an SPHS case before 2003. Of 47 SPHS cases, 37 (79%) had a confirmed diagnosis of leptospirosis. Single and paired samples were not obtained for testing from 2 and 8 of the 10 unconfirmed SPHS case-patients, respectively, all of whom died during hospitalization.

Among the 47 SPHS case-patients, 7 (15%) and 20 (42%) had pulmonary hemorrhage and respiratory insufficiency, respectively, at the time of hospitalization (Table 1). Pulmonary hemorrhage was identified in 19 (40%) patients only after endotracheal intubation (Table 2). Except for respiratory insufficiency, hemoptysis, and oliguria, SPHS case-patients had similar clinical manifestations to those of non-SPHS patients at the time of initial evaluation (Table 1). However, 7 (15%) and 16 (34%) of the 47 SPHS patients did not have signs of jaundice and acute renal insufficiency, respectively. Respiratory failure developed in all SPHS patients. Acute lung injury was documented in 25 (76%) of the 33 patients for whom arterial blood gas measurements were obtained (Table 2). Although patients received supportive care with mechanical ventilation (94%), dialysis (53%), and packed erythrocyte transfusion (60%), case-fatality rate for SPHS was 74% and significantly higher than that (12%) for non-SPHS leptospirosis (Table 1). By 2005, SPHS was the cause of 55% of the deaths among leptospirosis patients (Figure).

**Table 1:** Characteristics of leptospirosis patients identified during active surveillance in metropolitan Salvador, Brazil, from 2003 through 2005, according to presence of SPHS

Characteristics	With SPHS			Without SPHS		p value*
	No. responses	No. (%) or mean ± SD		No. responses	No. (%) or mean ± SD	
Age, y	44	37.6 ± 19.4		427	34.9 ± 14.5	0.57
Female sex	47	14 (30)		427	55 (13)	0.002
Clinical manifestations						
Days of symptoms before hospitalization	43	6.3 ± 3.6		419	6.1 ± 2.8	0.70
Respiratory insufficiency†	47	20 (42)		427	73 (17)	0.001
Hemoptysis	47	7 (15)		427	27 (6)	0.03
Jaundice	47	40 (85)		427	363 (85)	0.99
Total serum bilirubin, mg/dL	22	17.8 ± 13.4		140	13.9 ± 10.3	0.22
Oliguria	47	22 (47)		427	106 (25)	0.001
Blood urea nitrogen, mg/dL	33	121 ± 78		254	128 ± 79	0.60
Serum creatinine, mg/dL	33	3.9 ± 2.3		254	3.8 ± 2.7	0.64
Hypotension‡	42	12 (28)		387	85 (22)	0.33
Leukocyte count, 10^3^ cells/mm^3^	45	15.3 ± 8.0		422	14.4 ± 6.5	0.71
Hematocrit, %	23	31.8 ± 7.1		135	33.6 ± 6.1	0.11
Thrombocytopenia§	21	6 (29)		120	32 (27)	0.86
Therapeutic interventions						
Dialysis	47	25 (53)		425	89 (21)	0.001
Packed erythrocyte transfusion	47	28 (60)		424	42 (10)	0.001
Intensive care unit admission	47	44 (94)		424	112 (26)	0.001
Hospital outcome						
Death	47	35 (74)		427	49 (12)	0.001
Days of hospitalization for patients who died	35	3.2 ± 2.5		49	4.8 ± 5.9	0.91
Days of hospitalization for survivors	12	20.8 ± 15.0		378	9.1 ± 8.4	0.02
Confirmed case¶	47	37 (79)		427	351 (82)	0.56
Serovar Copenhageni as the presumptive infecting agent#	23	22 (96)		316	285 (90)	0.71

*The χ^2^ and Student *t* tests were used to evaluate for significant differences (p value <0.05) for proportions and continuous data, respectively. SPHS, severe pulmonary hemorrhagic syndrome; SD, standard deviation. †Respiratory rate >30 per min or use of supplemental oxygen therapy. ‡Systolic arterial blood pressure <90 mm Hg or use of vasoactive drugs. §Platelet count <50,000 cells/mm^3^. ¶The microscopic agglutination test (MAT) and immunoglobulin M ELISA were used for laboratory confirmation. #Proportions are shown for patients who had an MAT-confirmed diagnosis of leptospirosis and highest agglutination titers against *Leptospirosis interrogans* serovar Copenhageni.

**Table 2:** Pulmonary manifestations of patients with leptospirosis-associated severe pulmonary hemorrhagic syndrome (n = 47)

Characteristics*	No. responses	No. (%) or mean ± SD
Onset of massive hemoptysis		
During hospital admission	47	7 (15)
After hospitalization and before endotracheal intubation	47	21 (45)
During or after endotracheal intubation	47	19 (40)
Chest radiographic examination		
Bilateral alveolar infiltrates	24	21 (88)
Bilateral interstitial infiltrates	24	3 (12)
PaO_2_/FiO_2_, mm Hg	33	200 ± 155
Acute lung injury†	33	25 (76)
Acute respiratory distress syndrome‡	33	21 (64)

*Findings at initial evaluation. SD, standard deviation. †Defined as a ratio between arterial partial pressure of O_2_ and inspired O_2_ fraction (PaO_2_/FiO_2_) <300 mm Hg and the presence of bilateral alveolar or interstitial infiltrates on chest radiograph or bilateral crepitus on physical examination, without clinical evidence of increased left atrial pressure. ‡Defined as PaO_2_/FiO_2_ <200 mm Hg in addition to criteria for acute lung injury.

**Figure:**
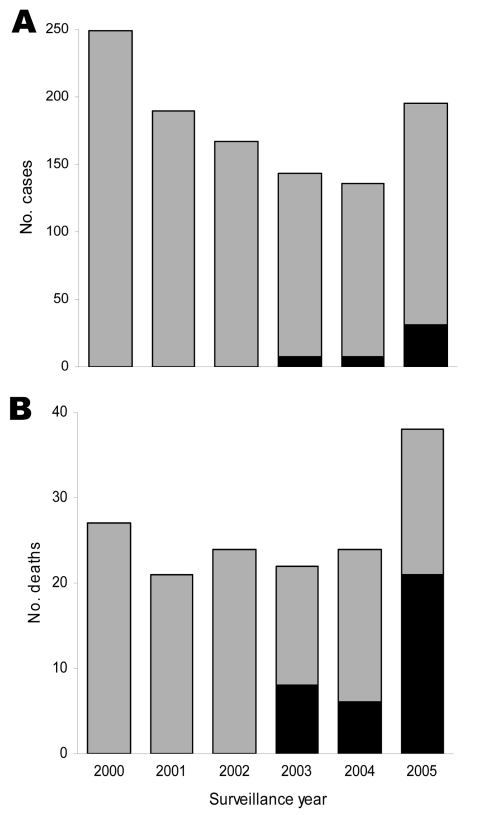
Active hospital-based surveillance for leptospirosis in Salvador, Brazil, 2000–2005, including total number of suspected leptospirosis cases (A) and deaths (B) among case-patients. Case-patients with and without severe pulmonary hemorrhagic syndrome are shown by black and gray bars, respectively.

The annual incidence of SPHS was 0.43 cases per 100,000 population, based on the 32 patients who resided within Salvador. The overall incidence of severe leptospirosis was 4.65 cases per 100,000 population (341 cases). SPHS cases occurred during the same winter period of seasonal rainfall during which non-SPHS leptospirosis cases were identified. All SPHS case-patients were residents of slum settlements from which non-SPHS case-patients were identified before and after the appearance of SPHS. Most SPHS case-patients were adults (mean age 37.6 ± 19.4 years) and male (70%) (Table 1). However, women had higher risk (30% vs. 17%, SPHS vs. non-SPHS leptospirosis; odds ratio 2.87, 95% confidence interval 1.36–5.98) of acquiring SPHS. A case-control investigation did not identify significant risk exposures for acquiring SPHS among leptospirosis cases. Serologic testing found that the highest agglutination titers were directed against *L. interrogans* serovar Copenhageni in 22 (96%) of 23 and 285 (90%) of 316 SPHS and non-SPHS cases, respectively, which were confirmed by MAT (Table 1). Culture isolation procedures were not implemented from 2001 through 2005, but an isolate was obtained from an SPHS patient in 2006. Serotyping procedures (*11*) identified it as *L. interrogans* serovar Copenhageni.

## Conclusions

There has been growing recognition of the importance of leptospirosis as the cause of SPHS (*1*–*3*). Yet except for a few outbreak situations (*4*–*6*), it remains unclear whether increased reporting represents enhanced detection of an under-recognized manifestation (*3*) or de novo emergence of this disease form. This investigation identified the appearance of SPHS in a region in which urban leptospirosis is endemic and active surveillance was in place. Prior under-recognition of this syndrome was unlikely because clinicians were aware of SPHS’s occurrence in other Brazilian cities (*7*,*8*,*12*). Laboratory confirmation of leptospirosis was obtained for 79% of the cases, indicating that SPHS was due to this disease rather than to other causes of hemorrhagic fever. Three years after the first case was identified, SPHS accounted for 19% of hospitalizations and 55% of the deaths from leptospirosis.

Our findings underscore the difficulties in identifying SPHS, even in the setting of heightened surveillance. The fatality rate was 74% among SPHS patients despite aggressive supportive care. Clinical parameters that could differentiate patients at risk of acquiring SPHS were not found during initial evaluation, thereby hampering attempts to implement timely triage procedures. Finally, pulmonary hemorrhage was only identified in 40% of the patients at the time of endotracheal intubation. Recognition of SPHS will therefore need to rely on a high index of suspicion in patients who have acute respiratory insufficiency.

That we did not identify environmental risk exposures for acquiring SPHS suggests a role for pathogen or host-specific factors. In Thailand, the recent sustained outbreak of leptospirosis was due to the widespread introduction of a *Leptospira* clone (*14*). Our findings suggest that the agent for SPHS in Salvador was *L. interrogans* serovar Copenhageni, which was also the cause of non-SPHS leptospirosis in this setting (*11*,*15*). Further isolation studies and genotyping analyses are needed to determine whether the appearance of SPHS is due to introduction or emergence of a clone that has enhanced virulence, within this serovar. Gender-specific factors, whether risk activities or host-susceptibility determinants, may have contributed to acquiring SPHS because women who with leptospirosis had twice the risk for this disease form. A large proportion of the world’s slum population resides in leptospirosis-endemic regions. Research is needed to elucidate the factors responsible for transmission of SPHS so that effective prevention can be identified.
